# McDonald versus Shirodkar cerclage technique in women requiring a prophylactic cerclage: a systematic review and meta-analysis protocol

**DOI:** 10.1186/s13643-021-01679-5

**Published:** 2021-05-01

**Authors:** Ashad Issah, Rosanna Diacci, Kimberley P. Williams, Anne-Marie Aubin, Liam McAuliffe, Jason Phung, Carol Wang, Panos Maouris, Craig E. Pennell

**Affiliations:** 1 School of Medicine and Public Health, The University of Newcastle, Callaghan, New South Wales Australia; 2 Mothers and Babies Research Centre, Hunter Medical Research Institute, New Lambton Heights, New South Wales Australia; 3 Maternity and Gynaecology John Hunter Hospital, New Lambton Heights, New South Wales Australia; 4 King Edward Memorial Hospital, Subiaco, Western Australia Australia

**Keywords:** Cervical, Stitch, Cerclage, McDonald, Shirodkar preterm birth

## Abstract

**Background:**

Preterm birth (PTB) is the leading cause of death in children under five years. Spontaneous preterm birth (SPTB) is the major cause of preterm delivery. The key risk factors for SPTB are women who have a short cervix and women who have had previous preterm birth. Cervical cerclage has been used for several decades and has shown to decrease rates of preterm birth. The most commonly used cerclage techniques were described by Shirodkar and McDonald, with no current consensus on the preferred technique. The objective of this review is to determine and compare the effectiveness of both techniques.

**Methods:**

Studies will be sourced from six electronic databases, as well as from experts in the field, reference lists, and grey literature. Eligible studies will include pregnant women, with a singleton or twin pregnancy, requiring a cervical cerclage, using either the Shirodkar or McDonald technique and run comparative analyses between the two techniques. Randomized control trials (RCT)s, non-randomized control trials, and cohort studies will be eligible. Two independent reviewers will conduct study screening at abstract and full-text level, data extraction and risk of bias assessment. Discrepancies will be resolved by a consensus third reviewer if required. Fixed-effects or random-effects models will be used where appropriate to synthesize results. Alternative synthesis methods will be investigated in instances where a meta-analysis is not appropriate, such as summarizing effect estimates, combining *P* values, vote counting based on direction of effect, or synthesis in narrative form.

**Discussion:**

This review will synthesize the evidence on both the Shirodkar and McDonald cerclage method, and will help clinicians and health services to determine and deliver best practice antenatal care that has the potential to make an impact on preterm birth.

**Systematic review registration:**

PROSPERO on 25 of May, 2020 with registration number CRD42020177386

## Introduction

### Description of the condition

Preterm birth (PTB), defined as birth prior to < 37 weeks gestation [1], remains a common complication of pregnancy (5–13%) [2] despite an increasing body of evidence surrounding its prevention. It is the leading cause of death in children under 5 years [3]. Short-term complications of PTB include respiratory distress syndrome, intraventricular hemorrhages, necrotizing enterocolitis, and retinopathy of prematurity, all of which contribute to the high perinatal morbidity and mortality rate of preterm infants [4].

Spontaneous preterm birth (SPTB) is the major cause of premature delivery(5). Compared to planned preterm birth, SPTB is regarded as a complex syndrome with multiple causes and includes deliveries after both preterm premature rupture of membranes (PPROM) and spontaneous preterm labor. The key risk factors of SPTB are a short cervix found on vaginal ultrasonography during pregnancy and a history of previous PTB [5–7]. In women with previous SPTB, singleton gestation, and cervical length less than 25 mm, cervical cerclage has been used for several decades and has been shown to decrease rates of PTB [8, 9].

### Description of the interventions

#### McDonald cerclage

In the McDonald approach, a suture is placed around the cervix in purse-string fashion and securely tied anteriorly. The McDonald approach requires no dissection into para-cervical tissues [10, 11].

#### Shirodkar cerclage

The modified Shirodkar technique, adapted from Shirodkar’s original described in 1955 [12], begins with a transverse incision made in the vaginal mucosa of the anterior cervix to allow for upward displacement of the bladder to avoid injury. A posterior incision is made in a similar fashion to avoid injury to the rectum. The lateral angles of the anterior and posterior incisions are then expanded with blunt fingertip dissection of the lateral cervix. A woven Mersilene tape on a large needle is then passed through the submucosal tunnel from anterior to posterior on both sides of the cervix. The lateral cervical mucosa at three and nine o’clock can be grasped with Allis clamp or ring forceps to facilitate suture placement. After the suture is placed on both sides of the cervix, the knot is tied in the posterior defect. The defects are then closed with an absorbable suture in a figure-of-eight fashion. In some instances, tape can be left extending through the closure so that it can be grasped and the suture exposed and cut when the patient goes into labor; otherwise, the remaining tape is buried under the cervical epithelium to reduce the chance of infection [10, 12]. For the purpose of this study, both the modified and the original approach described by Shirodkar will be included.

### The importance of this review

Despite numerous studies in the area, there are few high-quality papers, no systematic reviews or meta-analysis, and subsequently there is no clinical consensus on the preferred technique. Therefore, this review aims to appraise the body of literature and analyze the benefits of both techniques.

### Aim

The aim of this systematic review is to synthesize existing quantitative evidence comparing McDonald and Shirodkar cervical cerclage techniques to determine which of these has the best maternal and neonatal outcomes. Our proposed systematic review will answer this question in women requiring prophylactic cervical cerclage, does the McDonald or Shirodkar cerclage deliver better maternal and neonatal outcomes?

## Methods

### Registration

In accordance with the guidelines, our systematic review protocol was submitted to the International Prospective Register of Systematic Reviews (PROSPERO) on the 25th of May 2020 and was last updated on this date (registration number CRD42020177386). This review and meta-analysis will be completed with the recommendations of both Preferred Reporting Items for Systematic Reviews and Meta-Analyses Protocol (PRISMA-P) [13] (Appendix 1) and the Cochrane Handbook for Systematic Reviews of Interventions [14].

Information regarding registration can be accessed from http://www.crd.york.ac.uk/PROSPERO.

### Eligibility criteria

Eligibility of studies included in this systematic review will be based on pre-planned inclusion and exclusion criteria applied to each of the following domains: participant, exposure, comparator, study type, and outcome.

### Types of studies

This review will include randomized control trials, pseudorandomized control trials, non-randomized experimental control trials, and cohort studies. All papers included must compare the co-interventions, McDonald and Shirodkar cerclage. Papers which also include a control group will be included.

### Types of participants

This review will consider all studies that include pregnant women undergoing McDonald or Shirodkar cervical cerclage for prevention of PTB (before 37 weeks).

#### Intervention

For the purposes of this study, we will be considering the McDonald cerclage technique as the “intervention.”

#### Control

We will be considering the Shirodkar cerclage as the “comparator” or “control.”

#### Co-intervention

Studies will not be accepted where only one of the groups receives and additional intervention.

#### Outcomes

The primary outcome will be PTB of less than 37 weeks; subanalyses will be performed at 28, 32, 34, 35, and 36 weeks. Secondary outcomes will include neonatal survival; birthweight; Caesarean deliveries; days between cerclage insertion and delivery; PPROM; chorioamnionitis; cervical laceration and stenosis; Apgar score < 7; cases requiring repeat cerclage; fetal respiratory distress syndrome (FRDS); intraoperative rupture of membranes; and difference between cervical length before and after cerclage insertion. Outcomes of randomized control studies and observational studies will be reported separately. Additionally, subgroup analyses will be conducted for each outcome to compare the results of each study design.

### Search strategy

Electronic bibliographic databases will be searched for eligible, peer-reviewed literature including MEDLINE (Ovid); EMBASE (Ovid); PsycINFO (Ovid); Scopus; CINAHL (EBSCOhost); and Cochrane Library (Wiley). The reference lists of included studies will then be screened for potentially eligible studies. Studies recommended by experts in the field, references in academic textbooks, and grey literature will also be reviewed. There will be no restrictions on the length of the study follow-up period, country of origin, year of study, or language. Studies will be limited to human trials.

The search strategy will be developed through discussions with experts, academics in the field, pilot searches, and assessing systematic reviews on similar subjects. The search strategy will be focused on identifying papers with the two intervention techniques. Intervention search terms will include the name of the two procedures: “Shirodkar: and “McDonald,” combined with the type of procedure “cerclage,” “stitch,” or “suture” and the location of the intervention “cervical” or “cervix.” The term “rescue cerclage” was also found recurrently in the literature so “rescue” is also included as a search term.

Initially, a pilot search will be undertaken to identify further relevant keywords contained in titles and abstracts and subsequently a more thorough search will be undertaken. An academic librarian will be engaged to review keywords and medical subject headings (MeSH) in relevant databases. A more detailed database search strategy is described in Appendix 2.

### Data collection and analysis

#### Study selection

The titles and abstracts identified through all sources of the search will be downloaded to Endnote [15] where duplicates will be removed. Studies will then be uploaded to Covidence [16] and screened using the eligibility criteria described above. Studies that do not meet the criteria will be excluded. Full texts of the remaining articles will be sourced and screened. The remaining studies will be critically appraised and data extracted. At all levels of screening, two independent reviewers will conduct all screening and document exclusion. Any disagreements between reviewers at any level will be addressed at a meeting with an independent moderator. None of the reviewers are to be blinded to titles, study authors, publishing journals, or institutions.

#### Data management

The literature search will be uploaded to Covidence [16], an Internet-based software which enables collaboration among multiple reviewers during the process of study selection. Back-up copies of all texts will be kept in an Endnote library [15]. Screening questions, based on the inclusion criteria, will be developed by the reviewers.

#### Data collection process

Data from the eligible studies will be extracted by two independent reviewers and the data will then be compared. Any discrepancies will be moderated by a third reviewer. Data will be extracted using a standardized electronic form consistent with data collection items recommended by the Cochrane Handbook for Systematic Reviews of Interventions [14]. This process will be piloted prior to use. Any discrepancies in data extracted will be resolved by consensus between the two reviewers, or in consultation with a third reviewer if required. Reviewers extracting data will not be blind to author or journal information. Data will first be extracted through Covidence [16] before being transferred into RevMan data-analysis software [17].

The following data will be extracted:

- Study characteristics: authors; publication date; study design; country of study; sample size; confounding factors of participants; publication status; trial size; funding; and risk of bias information.
- Intervention characteristics: type of cerclage used; reason for cerclage including emergency/rescue cerclage; patient characteristics (maternal age, gravity, parity), and any co-interventions received.
- Outcomes: maternal, fetal and neonatal outcome data and definitions of each of the outcomes as described below.

### Outcomes and prioritization

#### Primary outcome

PTB of less than 37 weeks gestation with subanalysis at 28, 32, 34, 35, and 36 weeks. PTB is defined as stillbirth or live birth between 20 and 37 weeks gestation.

For papers which report outcomes as “greater than” *X* weeks data extractors will manually invert the figure to less than for integration.

#### Secondary outcomes

1. Neonatal survival: survival through the first 28 days post-delivery
2. Birthweight: weight of baby in grams post-partum prior to first feed
3. Caesarean deliveries: excluding planned or elective
4. Number of days between cerclage and delivery
5. PPROM: rupture of membranes prior to 37 weeks
6. Chorioamnionitis: clinical diagnosis or laboratory confirmed
7. Cervical laceration and stenosis: laceration diagnosed during delivery and stenosis diagnosed by physical examination or imaging
8. Apgar score < 7: defined using the 10-point Apgar scale [18] which awards up to 10 points for appearance, pulse, grimace, activity and respiration. Scores at either at 1, 5, or 10 min will be included
9. Fetal respiratory distress syndrome: clinical diagnosis or laboratory and/or imaging confirmed
10. Intra-operative membrane rupture at time of cerclage
11. Difference in cervical length before and after cerclage
12. Cases requiring repeat cerclage: women who had a cerclage in their current pregnancy which required a subsequent, repeat cerclage in the same pregnancy, at the discretion of the consulting obstetrician

#### Assessment of risk of bias

To facilitate the assessment of possible risk of bias for each study, we will assess each paper using the Cochrane Collaboration tool for assessing the risk of bias (ROBINS I and II) [19, 20] (Table 8.5.a in the Cochrane Handbook for Systematic Reviews of Interventions) [14]. Judgements using this tool will be made independently by two review authors with disagreements to be resolved by a third independent reviewer. We do not intend to exclude any study based on these scores but risk of bias will be taken into account when outcomes are assessed for impact with the Grading of Recommendations, Assessment, Development, and Evaluations (GRADE) [21].

#### Risk of bias in non-randomized studies of interventions

The Risk of Bias in Non-Randomized Studies of Interventions (ROBINS-I) [19] assessment tool will be used for non-randomized studies (observational studies). This tool includes seven bias domains pre-intervention, during intervention and post-intervention. The domains are: (1) confounding; (2) selection of participants; (3) classification of intervention; (4) deviation from interventions; (5) missing outcome data; (6) measurement of outcomes; and (7) selection of reported results overall. Risk of bias will be rated as no information, low risk, moderate risk, serious risk, and critical risk.

For randomized studies the Risk of Bias in Randomized Studies of Interventions (RoB2) [20] will be used. This tool involves five domains through which bias might be introduced into the result. These domains assess (1) the randomization process; (2) deviations from intended interventions; (3) missing outcome data; (4) measurement of the outcome, and (5) selection of the reported results.

#### Cochrane GRADE assessment

Quality of evidence for our primary outcome will be judged using the GRADE tool [21]. Evidence will be assessed in terms of risk of bias, consistency, directness, precision, and publication bias. With regard to GRADE, quality will be assessed as being one of four grades: (i) high—we are very confident that the true effect is close to that of the estimate of the effect; (ii) moderate—we are moderately confident in the effect estimate, and the true effect is likely to be close to the estimate of the effect, but there is a possibility that it is substantially different; (iii) low—our confidence in the effect estimate is limited, and the true effect may be substantially different from the estimate of the effect, and (iv) very low—we have very little confidence in the effect estimate, and the true effect is likely to be substantially different from the estimate of effect. Two independent reviewers will conduct the assessment, with discrepancies resolved through discussion and consensus between the two reviewers, or consultation with a third reviewer.

We will compute graphic representations of potential bias within and across studies using RevMan 5.1 (Review Manager 5.1) [17]. We will consider each item in the risk of bias assessment independently without an attempt to collate and assign an overall score.

### Data synthesis

A meta-analysis will be performed by pooling studies together using RevMan [17] and Covidence software [16]. The heterogeneity of data will be examined using forest plots. Further consideration of heterogeneity will be quantified by calculating the *I*^2^ value. An *I*^2^ of greater than or equal to 50% will be used to indicate substantial heterogeneity; should we find this, a random-effects model will be used, for all *I*^2^ less than 50% a fixed-effects model will be used. However, we will revert to a fixed-effects model for all outcomes with a small amount of studies (less than five) [22]. For reporting consistency between outcomes, we will make the McDonald intervention the reference set for all analyses, and this will standardize the direction of effect across all primary and secondary outcomes.

#### Measures of treatment effect

Where applicable, trial data will be combined and reported using meta-analyses using the standard estimation of (1) risk ratio (RR) and 95% confidence intervals (CI) for dichotomous outcome, and (2) mean differences (MDs) or standardized mean differences (SMDs) and 95% CIs for continuous outcome. We will use SMDs when studies report the same outcome and a comparable (but not identical) measure. In instances where a meta-analysis is not appropriate, alternative synthesis methods will be investigated as recommended by the Cochrane Handbook for Systematic Reviews of Interventions [14], such as summarizing effect estimates, combining *p* values, vote counting based on direction of effect, or synthesis in narrative.

#### Missing data

Where there is missing data, we will attempt to contact the study’s authors in order to obtain the data that is missing. If this is not possible, we will conduct sensitivity analysis excluding trials with high levels of missing outcome data (> 30%).

#### Meta-bias(es)

To determine if reporting bias is present, we will seek protocol papers for each study to determine if they were published prior to the study beginning and evaluate any discrepancies.

#### Sensitivity analysis

A sensitivity analysis will be conducted on the primary outcome. This will be performed by removing studies with an overall high risk of bias to examine their impact on the effect estimate.

## Discussion

This systematic review aims to determine the differences in effectiveness of the McDonald and Shirodkar cerclage techniques on neonatal and maternal outcomes. The review will be of value to clinicians, research policy-makers, the community, and those providing care to high risk obstetric women. The findings of this review will help to inform the development of evidence based guidelines and ultimately reduce the negative short- and long-term, health outcomes of preterm labor and birth for women and children. We do not anticipate any amendments to the present protocol. However, should any essential amendments be found to be necessary, they will be reported in the published review.

Heterogeneity in the papers included is often a limitation in systematic reviews. Specific sources for heterogeneity in this study included clinical variation in indication for cerclage, emergency vs elective caesarean section, the gestational age at time of cerclage, timing of removal of cerclage, previous obstetric history, and the variable reporting of secondary outcomes. In order to mitigate the effects of these factors on the outcomes evaluated, emergency, and rescue cerclage will be removed from this meta-analysis as these papers posed an unacceptable risk of bias. In addition, the random-effects model will be used for the analysis of studies with substantial heterogeneity (defined as *I*^2^ > 50%); outcomes with less the five studies will be, however, analyzed with the fixed-effects model regardless of *I*^2^.

Methodologically, this study is strengthened due to its rigid adherence to the PRISMA guidelines and the Cochrane Handbook for Systematic Reviews of Interventions. Furthermore, the papers in this study will be evaluated for its risk of bias and for its strength and quality of evidence presented using the ROBIN-1 and RoB-2 scores, and the GRADE assessment, respectively. The careful evaluation of the papers included ensures a quality and unbiased review of this study.

## Data Availability

Not applicable.

## Appendices

### Appendix 1

**Table 1:** PRISMA-P checklist

Section and topic	Item No	Checklist item	Line numbers of information reported
**Administrative information**			
Title:			
Identification	1a	Identify the report as a protocol of a systematic review	1–2
Update	1b	If the protocol is for an update of a previous systematic review, identify as such	N/A
Registration	2	If registered, provide the name of the registry (such as PROSPERO) and registration number	51–52, 108–110
Authors:			
Contact	3a	Provide name, institutional affiliation, e-mail address of all protocol authors; provide physical mailing address of corresponding author	5–24
Contributions	3b	Describe contributions of protocol authors and identify the guarantor of the review	364–367
Amendments	4	If the protocol represents an amendment of a previously completed or published protocol, identify as such and list changes; otherwise, state plan for documenting important protocol amendments	323–325
Support:			
Sources	5a	Indicate sources of financial or other support for the review	
Sponsor	5b	Provide name for the review funder and/or sponsor	356–359
Role of sponsor or funder	5c	Describe roles of funder(s), sponsor(s), and/or institution(s), if any, in developing the protocol	
**Introduction**			
Rationale	6	Describe the rationale for the review in the context of what is already known	93–104
Objectives	7	Provide an explicit statement of the question(s) the review will address with reference to participants, interventions, comparators, and outcomes (PICO)	99–104
**Methods**			
Eligibility criteria	8	Specify the study characteristics (such as PICO, study design, setting, time frame) and report characteristics (such as years considered, language, publication status) to be used as criteria for eligibility for the review	117–151, 158–160
Information sources	9	Describe all intended information sources (such as electronic databases, contact with study authors, trial registers or other grey literature sources) with planned dates of coverage	154–159
Search strategy	10	Present draft of search strategy to be used for at least one electronic database, including planned limits, such that it could be repeated	Appendix 2 456–465
Study records:			
Data management	11a	Describe the mechanism(s) that will be used to manage records and data throughout the review	187–191
Selection process	11b	State the process that will be used for selecting studies (such as two independent reviewers) through each phase of the review (that is, screening, eligibility and inclusion in meta-analysis)	176–185
Data collection process	11c	Describe planned method of extracting data from reports (such as piloting forms, done independently, in duplicate), any processes for obtaining and confirming data from investigators	193–202
Data items	12	List and define all variables for which data will be sought (such as PICO items, funding sources), any pre-planned data assumptions and simplifications	204–212
Outcomes and prioritization	13	List and define all outcomes for which data will be sought, including prioritization of main and additional outcomes, with rationale	214–238
Risk of bias in individual studies	14	Describe anticipated methods for assessing risk of bias of individual studies, including whether this will be done at the outcome or study level, or both; state how this information will be used in data synthesis	240–280
Data synthesis	15a	Describe criteria under which study data will be quantitatively synthesized	282–291
	15b	If data are appropriate for quantitative synthesis, describe planned summary measures, methods of handling data and methods of combining data from studies, including any planned exploration of consistency (such as *I*^2^, Kendall’s τ)	285–291
	15c	Describe any proposed additional analyses (such as sensitivity or subgroup analyses, meta-regression)	312–315
	15d	If quantitative synthesis is not appropriate, describe the type of summary planned	N/A
Meta-bias(es)	16	Specify any planned assessment of meta-bias(es) (such as publication bias across studies, selective reporting within studies)	308–310
Confidence in cumulative evidence	17	Describe how the strength of the body of evidence will be assessed (such as GRADE)	265–280

## Appendix 2: Search Strategy

1. Cervical OR Cervix OR Rescue
2. Stitch* OR cerclage OR Suture
3. 1 AND 2
4. McDonald
5. Shirodkar
6. 4 AND 5
7. 3 AND 6

For PubMed and Cochrane where MeSH headings are available the term “Cerclage, Cervical”[Mesh] will be added.

## Abbreviations

CI: Confidence interval
CINAHL: Cumulative Index of Nursing and Allied Health Literature.
GRADE: Grading of Recommendations, Assessment, Development and Evaluations
MD: Mean difference
MeSH: Medical subject headings
*P* value: Probability value
PRISMA-P: Preferred Reporting Items or Systematic Reviews and Meta-Analyses Protocol
PPROM: Preterm premature rupture of membranes
PROSPERO: International Prospective Register of Systematic Reviews
RCT: Randomized control trial
FRDS: Fetal respiratory distress syndrome
RevMan: Review Manager
ROBINS I: Risk of Bias in Non-Randomized Studies of Interventions
ROBINS II: Risk of Bias in Randomized Studies of Interventions
RR: Risk ratio
SPTB: Spontaneous preterm birth
SMD: Standardized mean difference
